# Circulating ADAMTS13 Levels Are Associated with an Increased Occurrence of Obstructive Sleep Apnea

**DOI:** 10.1155/2022/1504137

**Published:** 2022-03-29

**Authors:** Mengling Huang, Sheng Liu, Shuang Liu, Wanwan Wen, Yu Ning, Yifan Jia, Yunxiao Yang, Xiaolu Jiao, Weiping Zheng, Ming Zhang

**Affiliations:** ^1^Department of Cardiology, Beijing Anzhen Hospital, Capital Medical University, Beijing, China; ^2^Key Laboratory of Upper Airway Dysfunction-Related Cardiovascular Diseases, Beijing Anzhen Hospital, Capital Medical University, Beijing Institute of Heart, Lung, And Blood Vessel Diseases, Beijing 100029, China; ^3^Key Laboratory of Remodeling-Related Cardiovascular Diseases, Beijing Anzhen Hospital, Capital Medical University, Beijing Institute of Heart, Lung and Blood Vessel Diseases, Beijing 100029, China; ^4^Department of Clinical Laboratory, Beijing Anzhen Hospital, Capital Medical University, Beijing, China; ^5^Department of Cardiology, Shengli Clinical Medical College of Fujian Medical University, 134 East Street, Fuzhou, 350001 Fujian Province, China

## Abstract

**Background and Aims:**

Obstructive sleep apnea (OSA) is strongly associated with obesity, metabolic diseases, coronary artery disease (CAD), stroke, hypertension, and other disorders. This study assessed the relationship between circulating a disintegrin and metalloprotease with a thrombospondin type 1 motif, member 13 (ADAMTS13) levels and the presence of OSA.

**Materials and Methods:**

This cross-sectional study included a total of 223 patients. We used a powerful high-throughput multiplexed immunobead-based assay to detect circulating levels of ADAMTS13. The associations between circulating ADAMTS13 levels and OSA were evaluated by multivariate logistic regression analysis.

**Results:**

Circulating ADAMTS13 levels were significantly elevated in patients with OSA compared with controls (0.8 vs. 2.7 *μ*g/mL, respectively, *P* < 0.001). After adjusting for confounding factors, circulating ADAMTS13 levels were significantly independently associated with the presence of OSA (odds ratio = 9.96, 95% confidence interval (CI) =4.11–24.13, *P* < 0.001). Furthermore, circulating ADAMTS13 levels showed discriminatory accuracy in assessing the presence of OSA (area under the curve: 0.87, 95% CI 0.81–0.93, *P* < 0.001).

**Conclusion:**

Circulating ADAMTS13 levels were significantly correlated with the presence of OSA. ADAMTS13 may therefore function as a novel biomarker for monitoring the development and progression of OSA.

## 1. Introduction

Obstructive sleep apnea (OSA) is a relatively common sleep disorder characterized by repetitive collapse of the upper airway leading to chronic intermittent hypoxia (CIH) and sleep disruption, sleep fragmentation, and excessive daytime sleepiness [1]. It affects at least 10% of adults worldwide [2] and has a growing prevalence because of the obesity epidemic. Patients with OSA are at high risk of hypertension, metabolic diseases, coronary artery disease (CAD), stroke, and other disorders [3, 4].

Several studies have reported multiple risk factors for OSA, including obesity, sex, fluid retention, adenotonsillar hypertrophy, and smoking [5–9]. However, the pathogenesis of OSA is not fully understood, and current methods of diagnosis and treatment are inadequate. Clinical practice guidelines from the American Academy of Sleep Medicine (AASM) propose overnight polysomnography (PSG) as a means of screening and diagnosing OSA in adults with suspected OSA. However, PSG is expensive, labor-intensive, time-consuming, and impractical for the clinical evaluation of large at-risk populations [1]. Therefore, a comprehensive evaluation of the molecular indexes of OSA will help precisely detect and diagnose this sleep disorder in clinical practice.

A disintegrin and metalloprotease (reprolysin type) with a thrombospondin type 1 motif (ADAMTS) is a family of proteins with characteristic ADAM-like protease domains, disintegrin-like cysteine-rich domains, and no transmembrane domains that are in the extracellular matrix (ECM) [10, 11]. ADAMTS, member 13 (ADAMTS13) is a member of the family that circulates within the blood and reduces the activity of von Willebrand factor (vWF) in platelet adhesion and aggregation by cleaving prothrombotic vWF multimers [12–14]. It is primarily synthesized by stellate cells of the liver and vascular endothelial cells and proteolyzes ultralarge vWF multimers (ULvWF) at the Tyr1605-Met1606 bond in the A2 domain [15]. Low ADAMTS13 levels and activity are associated with an increased risk of metabolic diseases, including ischemic stroke and myocardial infarction, as well as kidney disease [16]. They also regulate obesity, inhibit inflammatory reactions, matrix degradation, and tissue remodeling, and promotes angiogenesis and atherosclerosis [17–22], which are associated with OSA. However, the association of ADAMTS13 with OSA remains unexplored. Therefore, the present study examined whether circulating ADAMTS13 levels are associated with the presence of OSA.

## 2. Methods

### 2.1. Study Design and Participants

We conducted a cross-sectional study at the Sleep Center of Beijing Anzhen Hospital between October 2019 and September 2021. The study was approved by the Chinese Clinical Trial Registry (no. ChiCTRROC-17011027), and all participants provided their written informed consent. The study protocol was approved by the Medicine Ethics Committee of Beijing Anzhen Hospital and adhered to the principles laid out in the Declaration of Helsinki.

A total of 263 patients who underwent an overnight full PSG at Beijing Anzhen Hospital were originally eligible for the study. Forty-seven of these patients with the following underlying factors were excluded: <18 years of age; a history of liver disease, chronic kidney disease, cancer, acute infectious diseases, chronic obstructive pulmonary disease, bronchial asthma, or interstitial lung disease; pregnancy; and receiving therapy for OSA. Therefore, 223 subjects were included in the study. The Epworth Sleepiness Scale (ESS) was used to identify sleepiness.

**Figure 1:**
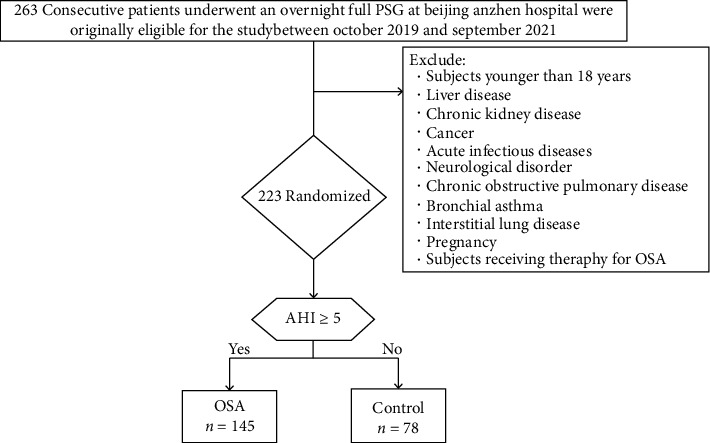
Study flow. 223 participants were consecutively enrolled including 78 healthy controls and 145 OSA patients.

Demographic characteristics and patient clinical history were recorded. Weight (kg), height (m), and blood pressure (mmHg) were measured in the morning after the PSG examination. The body mass index (BMI) (kg/m^2^) was calculated as weight divided by height squared. Neck circumference (cm) was measured between the midcervical spine and midanterior neck in the standing position [23, 24]. Current smokers were defined as participants who were currently smoking or had stopped smoking less than 1 year before enrollment in this study. CAD was defined if any of the following characteristics were observed: a history of physician-diagnosed CAD or the use of medications (oral antiplatelet drugs (aspirin and/or clopidogrel) along with statins) for CAD; angiographic CAD was defined as the presence of ≥50% luminal stenosis in at least one major coronary artery [25]. Diagnoses of diabetes were based on American Diabetes Association criteria [26]. The study design is described in detail in Figure 1.

### 2.2. Sleep Data

All participants included in the study underwent PSG in a sleep laboratory under standardized conditions [27, 28]. All sleep studies were scored by experienced physicians, after the removal of movement and technical artifacts, according to the standard criteria defined by the AASM [29]. Hypopnea was defined as ≥3% oxygen desaturation sustained for ≥10 s, while apnea was defined as a complete absence of airflow or an airflow decrease ≥ 90% relative to the baseline amplitude, persisting for ≥10 s. The number of hypopneas plus apneas per h of sleep was defined as AHI. We recorded the AHI, lowest oxygen saturation (lowest SaO_2_), mean oxygen saturation (MSaO_2_), percentage of cumulative time with oxygen saturation below 90% (CT90), and arousal index. OSA was classified as mild OSA (5 ≤ AHI < 15 events/h), moderate OSA (15 ≤ AHI < 30 events/h), and severe OSA (AHI ≥ 30 events/h).

### 2.3. Blood Sampling

All blood samples were collected after the participants had fasted overnight. Samples were centrifuged for 10 min at 3000 rpm at 4°C; then, plasma samples were stored at –80°C before analysis. Serum uric acid, high-sensitivity C-reactive protein, triglycerides (TG), total cholesterol (TC), low-density lipoprotein (LDL), high-density lipoprotein (HDL), fasting blood glucose (FBG), *γ*-glutamyl transferase (GGT), alanine aminotransferase (ALT), aspartate aminotransferase (AST), creatinine, and other routine serum biochemical parameters were measured in a biochemical analyzer (Hitachi-7600, Tokyo, Japan) using blinded quality control specimens in the Department of Biochemistry at Beijing Anzhen Hospital.

### 2.4. Magnetic Luminex Assays

This study uses the method of Wen et al., and the method description partly reproduces their wording [30]. The Magnetic Luminex® assay is a magnetic bead-based antibody microarray founded upon the sandwich immunoassay principle, which is used to assess the levels of biomarkers in a single sample [31, 32]. We used a panel of unique, custom-made magnetic bead cytokines, including ADAMTS13, to screen biomarkers capable of predicting OSA (R&D Systems, Inc., Minneapolis, MN, USA) [33]. The assay was performed by a trained investigator who was blinded to the clinical status of the subjects. To ensure the accuracy and validity of the results, we evaluated the Luminex multiplex assay system by standard curve and intra-assay variability, with intra-assay coefficient of variation (CV) < 10% being acceptable [34]. In our study, the intra-assay CV of the standard was <4.0%. The intra-assay CV of ADAMTS13 is shown in Supplemental Table S1. The absorbance of each well was determined at 450 nm. Circulating ADAMTS13 levels were determined simultaneously by the Human Magnetic Luminex Screening Assay in accordance with the manufacturer's instructions. The concentrations of each cytokine were determined using the Bio-Rad Bio-Plex 200 system (Bio-Rad Laboratories, Inc., Hercules, CA, USA). Data were analyzed by 5-parameter curve fitting using Bio-Plex Manager software, version 6.1.1 (Bio-Rad).

### 2.5. Statistical Analysis

Continuous variables are presented as means ± standard deviation or medians and interquartile ranges. According to the standard OSA diagnostic criteria, the subjects were divided into the following two groups: OSA, defined as an apnea-hypopnea index (AHI) score of ≥5 events per h, and non-OSA. Categorical variables are described in terms of absolute and relative frequencies. Continuous variables were compared by the independent Student *t*-test, one-way analysis of variance (ANOVA), or nonparametric Mann–Whitney *U* or Kruskal–Wallis *H* tests. Pearson's chi-square test or Fisher's exact test was performed to compare categorical variables. Spearman's correlation coefficient was used to analyze the correlations of continuous variables, such as the relationship between circulating ADAMTS13 levels and OSA-related variables.

The association between circulating ADAMTS13 levels and OSA was determined by multivariate logistic regression analyses (adjusted for age, sex, BMI, CAD, and smoking). Multiple linear regression analyses (forced entry method) were performed to assess the influence of variables (age, sex, BMI, and other variables with *P* < 0.05 in univariate analysis) on circulating ADAMTS13 levels and AHI levels. ADAMTS13 levels were log-transformed in the regression analysis. To evaluate the predictive power of the identified predictors of OSA, we used receiver operating characteristic (ROC) curves and, in particular, the associated area under the curve (AUC). A *P* value < 0.05 was considered statistically significant. Statistical analyses were performed using SPSS 20.0 software (IBM Corp., Armonk, NY, USA).

## 3. Results

### 3.1. Baseline Clinical Characteristics of the Study Population

The present study included 145 patients with OSA and 78 control subjects. Demographic and sleep data of the participants are shown in Table 1. PSG findings showed significant differences between OSA patients and controls with respect to AHI, ESS, MSaO_2_ (%), lowest SpO_2_ (%), CT90 (%), and arousal index (events/h).

**Figure 2:**
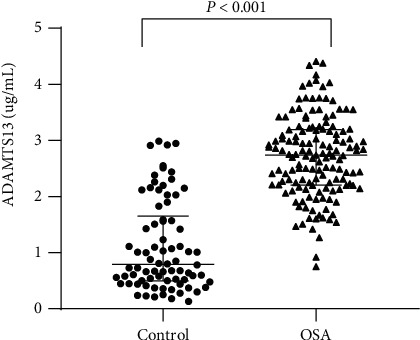
Circulating ADAMTS13 levels showing significant elevation in patients with OSA compared with controls (*P* < 0.001).

Laboratory data of the participants are shown in Table 2. OSA patients showed significantly elevated BMI, systolic blood pressure (SBP), diastolic blood pressure (DBP), TC, FBG, ALT, GGT, creatinine, and uric acid levels, as well as neck circumference compared with controls (*P* < 0.05). Additionally, circulating ADAMTS13 levels were significantly increased in OSA patients compared with controls (0.8 (range, 0.5–1.7) *μ*g/mL vs. 2.7 (range 2.2–3.2) *μ*g/mL, respectively, *P* < 0.001) (Figure 2).

### 3.2. Correlation of Circulating ADAMTS13 Levels with Clinical and Biological Parameters

**Figure 3:**
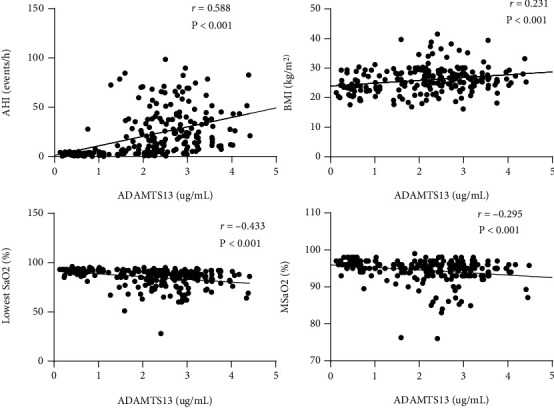
Scatterplots of circulating ADAMTS13 levels vs. AHI, lowest SaO_2_, MSaO_2_, and BMI.

As shown in Figure 3 and Supplemental Table S2, Spearman's correlation was used to explore the association between circulating ADAMTS13 levels with clinical and biological parameters. The data indicated that circulating levels of ADAMTS13 in all individuals were significantly correlated with age (*r* = 0.153, *P* < 0.001), BMI (*r* = 0.231, *P* < 0.001), sex (*r* = –0.228, *P* < 0.05), AHI (*r* = 0.588, *P* < 0.001), lowest SaO_2_ (*r* = –0.433, *P* < 0.001), SBP (*r* = 0.162, *P* < 0.05), arousal index (*r* = 0.251, *P* < 0.001), FBG (*r* = 0.237, *P* < 0.001), TC (*r* = 0.160, *P* < 0.05), HDL (*r* = –0.315, *P* < 0.001), and ALT (*r* = 0.229, *P* < 0.001).

### 3.3. Association of Circulating ADAMTS13 Levels with the Presence of OSA

As shown in Supplemental Table S3, univariate analysis demonstrated that circulating ADAMTS13 levels were associated with OSA (odds ratio (OR) = 10.695, 95%confidence interval (CI) = 5.933-19.280, *P* < 0.001). As shown in Table 3, the association of circulating ADAMTS13 levels with the presence of OSA was investigated in different models of binary logistic regression. After adjustment for age, sex, BMI, smoking habit, SBP, DBP, TG, TC, LDL, HDL, ALT, AST, GGT, uric acid, creatinine, and FBG, we found that the circulating ADAMTS13 level was an independent risk factor for OSA (OR = 13.305, 95%CI = 5.338-33.161, *P* < 0.001). As shown in Table S4

, with age, sex, CAD, BMI, smoking habit, SBP, DBP, TG, TC, LDL, HDL, ALT, AST, GGT, uric acid, creatinine, and FBG as covariates, circulating ADAMTS13 levels positively correlated with AHI, which represents the severity of OSA (*β* = 7.335/100 ng ADAMTS13, 95%CI = 4.285-10.385, *P* < 0.001).

### 3.4. ROC Curve Analysis for Circulating ADAMTS13 Levels in Discriminating OSA

**Figure 4:**
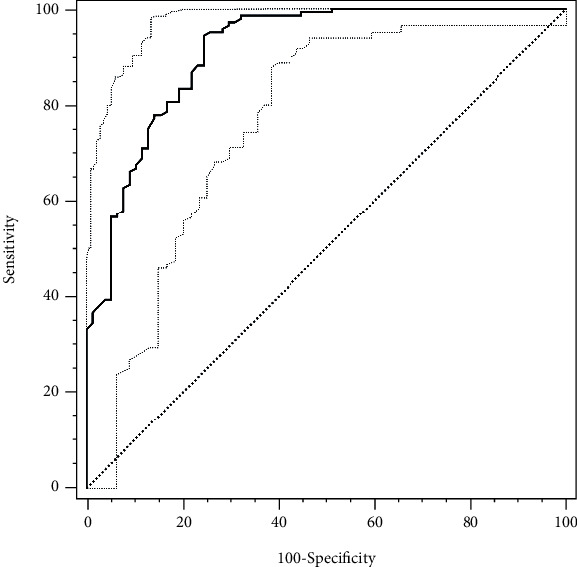
ROC curve analysis of ADAMTS13 for predicting OSA. Area under the curve (AUC) = 0.91 and 95% CI: 0.87, 0.95. *P* < 0.001.

**Figure 5:**
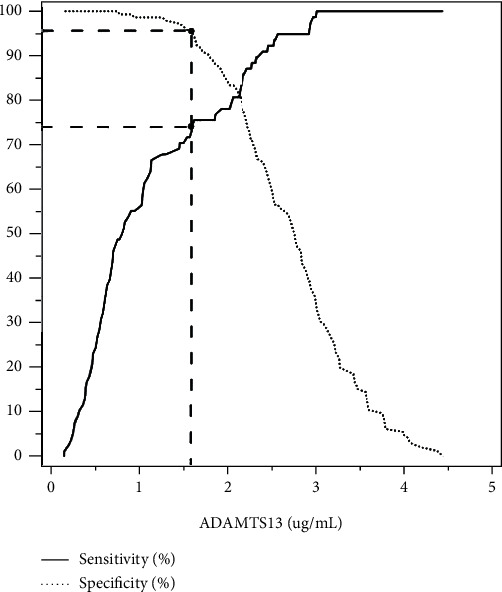
Sensitivity and specificity for ADAMTS13 in predicting OSA. A cutoff value of 1.58 *μ*g/mL corresponded to a sensitivity of 95.17% and specificity of 74.36%.

We next used ROC analysis to evaluate the use of circulating ADAMTS13 levels in discriminating between OSA and severe OSA (Figure 4). The ability of the AUC, based on circulating ADAMTS13 levels, to discriminate the presence of OSA was 0.91 (95%CI = 0.87–0.95, *P* < 0.001), indicating that circulating ADAMTS13 levels are a potential biomarker of OSA. The optimal cutoff value of circulating ADAMTS13 levels for the identification of OSA was 1.58 *μ*g/mL with a corresponding sensitivity of 95.17% and specificity of 74.36% (Figure 5).

## 4. Discussion

In the present study, we detected significantly higher circulating ADAMTS13 levels in patients with OSA compared with control individuals, even after adjusting for other known risk factors. Moreover, circulating ADAMTS13 levels were markedly correlated with AHI in our study, while ROC analysis suggested that they may be important discriminative indicators of OSA, especially in cases of severe OSA. These results reveal circulating ADAMTS13 levels to be a promising biomarker for the occurrence of OSA.

Recently, some association between the ADAMTS (a disintegrin and metalloproteinase (ADAM) with thrombospondin motifs) family's proteinases and OSA has been implicated [35]. In our study, the circulating ADAMTS13 levels were increased in OSA patients. A previous cross-sectional study investigating the association between OSA and circulating ADAMTS13 levels [36] found no significant difference in ADAMTS13 levels between 58 OSA patients (receiving nasal CPAP therapy) and 25 sleep controls, which indicated the CPAP therapy may alleviate the increase in the circulating ADAMTS13 levels by OSA. The mechanism underlying the association of circulating ADAMTS13 levels with OSA remains unclear. Nevertheless, our findings show that the association is robust to adjustments for obesity, cardiovascular disease, metabolic diseases, and hypertension. ADAMTS13 levels have previously been reported to be associated with obesity (BMI) and blood lipid levels (cholesterol, TG, and HDL) [37, 38], while obesity is the strongest risk factor for the development of OSA [1]. Moreover, ADAMTS13 synthesis is significantly enhanced in mice with obesity and/or hypercholesterolemia compared with control animals [37], but the increase is primarily found in male mice, suggesting a sex-dependent regulatory mechanism. Lee observed a strong positive correlation between cholesterol and ADAMTS13 levels in wild-type mice, but their findings did not support a functional role for ADAMTS13 in obesity nor in associated angiogenesis or inflammation, at least in mice [39].

In the present study, we found that ADAMTS13 levels were positively associated with obesity and TC and that this association remained after adjusting for BMI and TC. It is important that ADAMTS13 is found to be associated with AHI (*r* = 0.588, *P* < 0.001) and lowest SaO_2_ (*r* = –0.433, *P* < 0.001). There was also a study in which Ferreira et al. demonstrated that patients with higher ADAMTS13 were more likely to have Cheyne‐Stokes respiration (CSR) ≥ 20% [40]. A lot of researches indicated that hypoxia may affect endothelial damage and angiogenesis [41–43], while the inherent mechanism of ADAMTS13 is unclear. Recent research suggests that this conformational change also increases the ability of ADAMTS13 to break down other proteins such as fibrinogen [44]. Therefore, the observed association between ADAMTS13 activity and OSA might be explained by the interaction of ADAMTS13 with one or more currently unknown proteins. The association could be explained by pathways responding to ADAMTS13. There are some preliminary evidences that ADAMTS13 upregulates vascular endothelial growth factor (VEGF), a protein involved in angiogenesis [45–47]. Abu et al. found that tissue inhibitor of matrix metalloproteinase-3 (TIMP-3) (an efficient inhibitor of several members of the ADAM and ADAMTS) had powerful regulatory effects on anti-inflammatory and antiangiogenic activities [48]. Meanwhile, OSA is associated with angiogenesis [49]. Mun et al. revealed that rats with an intermittent hypoxic brain condition stimulated vascular proliferation for spontaneous recovery through VEGF elevation [49].

Previous studies showed that age was an independent risk factor for OSA and that most individuals with OSA were male [50]. Similarly, we observed more males in the OSA group than in the control group; therefore, to avoid confounding effects, we adjusted for age and sex in this study. Obesity also plays an important role in OSA. Weight loss reduces anti-ADAMTS13 autoantibodies and improves inflammatory and coagulative parameters in obese patients [51]. However, although ADAMTS13 is associated with BMI, it might not affect angiogenesis despite being a known factor of lipid and glucose metabolism [52]. We found that patients with OSA had higher BMI and TG levels than non-OSA subjects. Recent studies established OSA and chronic intermittent hypoxia (CIH) as risk factors for nonalcoholic fatty liver disease (NAFLD) [53, 54], and CIH was shown to be independently associated with NAFLD. ADAMTS13 is mainly synthesized and secreted by liver endothelium cells [10], and we observed an association between ADAMTS13 and ALT levels in this study. Therefore, to avoid confounding effects, we also adjusted for BMI, TC, TG, HDL, FBG, and ALT.

Despite the fact that care was taken to avoid bias in this study, such as the trained investigator blinded to the subjects' clinical status performing the Luminex assays, adjustments in the statistical analysis for the confounding factors, and consecutive recruitment of subjects, our study still had some limitations. First, it was a cross-sectional study with a relatively small sample size, which meant that it could only show associations, not causality. Second, we did not evaluate the effect of continuous positive airway pressure therapy or upper airway surgery on plasma ADAMTS13 expression. Third, potential false-positive results have occurred despite multiple corrections. Therefore, prospective cohort studies are needed to confirm the variants and associations identified in our study. Finally, the controls of this study were enrolled from the Otolaryngological Department of our hospital rather than the general population.

In conclusion, an early diagnosis of OSA is crucial to start therapy that will improve the outcome of OSA-related cardiovascular complications and reduce mortality and treatment costs [30, 55]. The diagnosis of OSA-induced subclinical disease before the occurrence of symptoms may also play a role in initial screening for OSA [30, 56]. Therefore, a comprehensive understanding of the molecular indexes underlying OSA is important to help precisely detect and diagnose this disorder in clinical practice. We herein demonstrated that circulating ADAMTS13 levels can be used to show the presence of OSA with a high degree of sensitivity and specificity. Future studies are needed to highlight the inherent mechanisms of OSA and the predictive value of ADAMTS13 in the outcome of patients with OSA. We identified circulating ADAMTS13 levels as a novel independent marker of incident OSA. ADAMTS13 can be a marker to predict the occurrence of OSA. Further research is necessary to confirm this association and to elucidate the biological mechanism underlying this association.

## Figures and Tables

**Table 1:** Comparison of demographic and sleep data between OSA patients and controls.

Variables	Controls (*n* = 78)	OSA (*n* = 145)	*P* value
Anthropometric characteristics			
Age (years)	49.6 ± 15.5	53.1 ± 12.2	0.798
Male, *n* (%)	49 (62.8%)	119 (82.1%)	0.002
BMI (kg/m^2^)	23.7 ± 3.1	27.5 ± 4.0	<0.001
Current smoker, *n* (%)	26 (33.3%)	57 (39.3%)	0.318
Diabetes mellitus, *n* (%)	10 (12.8%)	21 (14.5%)	0.476
Hypertension, *n* (%)	24 (30.7%)	83 (57.2%)	<0.001
Hypercholesterolemia, *n* (%)	8 (10.3%)	24 (16.6%)	0.304
CAD, *n* (%)	13 (16.7%)	69 (47.6%)	<0.001
SBP (mmHg)	120.0 (112.5-134.0)	124.5 (117.0-135.3)	0.027
DBP (mmHg)	70.0 (63.2-83.5)	79.0 (71.0-88.0)	0.011
Sleep data			
Neck circumference (cm)	37.9 ± 3.9	40.7 ± 4.2	<0.001
AHI (events/h)	2.7 (1.6-3.9)	29.1 (16.7-59.4)	<0.001
ESS	0 (0-7)	9 (5-13)	<0.001
MSaO_2_ (%)	96.0 (95.0-97.0)	95.0 (93.0-95.2)	<0.001
Lowest SaO_2_ (%)	92.0 (91.0-93.3)	81.0 (74.0-87.8)	<0.001
CT90% (%)	0 (0-0.15)	2.8 (0.0-17.6)	<0.001
Arousal index (events/h)	4.1 (0.0-9.6)	12.6 (6.7-40.0)	<0.001

Data are presented as *n* or *n* (%), mean ± SD, or median (interquartile range, IQR), unless otherwise stated. OSA: obstructive sleep apnea; BMI: body mass index; CAD: coronary artery disease; SBP: systolic blood pressure; DBP: diastolic blood pressure; AHI: apnea-hypopnea index; ESS: Epworth Sleepiness Scale; MSaO_2_: mean oxygen saturation; lowest SaO_2_: lowest oxygen saturation; CT90: percentage of cumulative time with oxygen saturation below 90% during sleep time.

**Table 2:** Comparison of laboratory results between OSA patients and controls.

Variables	Controls (*n* = 48)	OSA (*n* = 145)	*P* value
Platelet (G/L)	216.0 (173.5-262.8)	215.5 (190.0-249.5)	0.885
Leukocyte (G/L)	5.6 (4.8-7.5)	6.2 (5.5-7.1)	0.135
FBG (mmol/L)	5.1 (4.8-5.4)	5.5 (5.1-6.1)	0.004
Uric acid (*μ*mol/L)	333.5 ± 86.6	386.9 ± 95.9	<0.001
Urea nitrogen (mmol/L)	4.5 (4.1-5.9)	5.1 (4.3-6.4)	0.054
Creatinine (*μ*mol/L)	66.0 ± 12.2	71.7 ± 22.1	<0.001
Creatine kinase (U/L)	102.4 ± 50.1	116.5 ± 108.7	0.402
Total cholesterol (mmol/L)	4.2 (3.6-5.0)	4.7 (3.7-5.4)	<0.001
Triglyceride (mmol/L)	1.5 (1.0-2.0)	1.5 (1.1-2.0)	0.144
HDL (mmol/L)	1.3 (1.2-1.4)	1.1 (1.1-1.2)	0.042
LDL (mmol/L)	2.6 (1.2-4.2)	2.1 (1.6-2.9)	0.275
hsCRP (mg/L)	0.9 (0.3-3.4)	1.4 (0.6-3.5)	0.094
GGT (U/L)	26.0 (16.8-36.8)	33.0 (22.0-48.3)	0.001
ALT (U/L)	21.5 (13.0-30.5)	26.0 (17.0-36.0)	0.024
AST (U/L)	20.0 (18.0-24.3)	21.0 (18.0-28.0)	0.162
ADAMTS13 (*μ*g/mL)	0.8 (0.5-1.7)	2.7 (2.2-3.2)	<0.001

Data are presented as *n* or *n* (%), mean ± SD, or median (interquartile range, IQR), unless otherwise stated. FBG: fasting blood glucose; HDL: high-density lipoprotein; LDL: low-density lipoprotein; FBG: fasting blood glucose; hsCRP: high-sensitivity C-reactive protein; GGT: *γ*-glutamyl transferase; ALT: alanine aminotransferase; AST: aspartate aminotransferase; ADAMTS13: a disintegrin and metalloprotease with a thrombospondin type 1 motif, member 13.

**Table 3:** Multivariate logistic regression analysis of circulating ADAMTS13 levels and OSA.

	Unadjusted		Model 1		Model 2	
	OR (95% CI)	*P* value	OR (95% CI)	*P* value	OR (95% CI)	*P* value
ADAMTS13(per ng/mL increase)	10.695 (5.933-19.280)	<0.001	11.105 (5.753-21.434)	<0.001	13.305 (5.338-33.161)	<0.001

Model 1: adjusted for age, sex, BMI, CAD, and smoker. Model 2: adjusted for model 1+SBP, DBP, TG, TC, LDL, HDL, ALT, AST, GGT, uric acid, creatinine, and FBG. CI: confidence interval; OR: odds ratio; BMI: body mass index; CAD: coronary artery disease; DBP: diastolic blood pressure; SBP: systolic blood pressure; FBG: fasting blood glucose; GGT: *γ*-glutamyl transferase; HDL: high-density lipoprotein; LDL: low-density lipoprotein; LDL: low-density lipoprotein cholesterol; ALT: alanine aminotransferase; AST: aspartate aminotransferase; OSA: obstructive sleep apnea; ADAMTS13: a disintegrin and metalloprotease with a thrombospondin type 1 motif, member 13.

## Data Availability

The data used to support the findings of this study are included within the article.

## Supplementary Materials

Supplementary Materials Table S1: evaluation of Luminex assay standard curves and intra-assay variability for circulating ADAMTS13 levels determined using the Luminex. Table S2: correlation of ADAMTS13 with clinical and laboratory variables. Table S3: associations between clinical or biochemical variables and OSA. Table S4: multivariate linear regression analysis of circulating ADAMTS13 levels and AHI.
