# Supplementary material for: Circulating ADAMTS13 Levels Are Associated with an Increased Occurrence of Obstructive Sleep Apnea
Source: Dis Markers. 2022 Mar 29;2022:1504137. doi: 10.1155/2022/1504137 (PMC8983172; doi:10.1155/2022/1504137)
Supplement: Supplementary Materials — Table S1: evaluation of Luminex assay standard curves and intra-assay variability for circulating ADAMTS13 levels determined using the Luminex. Table S2: correlation of ADAMTS13 with clinical and laboratory variables. Table S3: associations between clinical or biochemical variables and OSA. Table S4: multivariate linear regression analysis of circulating ADAMTS13 levels and AHI. [file 1504137.f1.docx]

**Supplemental Files**

**Table S1** Evaluation of Luminex assay standard curves and intra-assay variability for circulating ADAMTS13 levels determined using the Luminex

| Cytokines | LoB(pg/L) | LoD(pg/L) | LLOQ(pg/L) | ULoQ(pg/L) | Intra-assay(CV%) |
| --- | --- | --- | --- | --- | --- |
| AMDMTS13 | 6058.7 | 8032.3 | 20038 | 4530860 | 0.79-2.16 |

LoB: Limit of Blank; LoD: Limit of Detection; LLoQ: Lower Limit of Quantitation; ULoQ: Upper Limit of Quantitation; CV: Coefficient of variation; ADAMTS13, a disintegrin and metalloprotease with a thrombospondin type 1 motif, member 13.

**Table S2** Correlation of ADAMTS13 with clinical and laboratory variables.

| Variables | rho |  |  |
| --- | --- | --- | --- |
| Age (years) | 0.153* |  |  |
| BMI (kg/m^2^) | 0.231** |  |  |
| Male (female vs male) | -0.228* |  |  |
| AHI (events/h) | 0.588** |  |  |
| Lowest SaO_2_ (%) | -0.433** |  |  |
| CT90% (%) | 0.262** |  |  |
| Arousal index (events/h) | 0.251** |  |  |
| SBP (mm Hg) | 0.162* |  |  |
| DBP (mm Hg) | 0.088 |  |  |
| hs-CRP (mg/L) | 0.135 |  |  |
| FBG (mmol/L) | 0.237** |  |  |
| TC (mmol/L) | 0.160* |  |  |
| HDL (mmol/L) | -0.315** |  |  |
| ALT (U/L) | 0.229** |  |  |

Spearman’s rho was used for correlations of ADAMTS13 with clinical and laboratory variables.

BMI, body mass index; AHI, apnea-hypopnea index; Lowest SaO_2_, lowest oxygen saturation; CT90, percentage of cumulative time with oxygen saturation below 90% during sleep time; SBP, systolic blood pressure; DBP, diastolic blood pressure; hsCRP, high-sensitivity C-reactive protein; FBG, Fasting blood glucose; TC, Total cholesterol; ADAMTS13, a disintegrin and metalloprotease with a thrombospondin type 1 motif, member 13.

*P < 0.05, **P < 0.001.

**Table S3** Associations between clinical or biochemical variables and OSA.

|  | OR | 95%CI | P-value |
| --- | --- | --- | --- |
| Age (year) | 1.001 | 0.981 – 1.022 | 0.892 |
| Male (female vs male) | 2.709 | 1.450 – 5.062 | **0.002** |
| BMI (kg/m^2^) | 1.280 | 1.167 – 1.404 | **＜0.001** |
| CAD n (%) | 0.427 | 0.236-0.772 | **0.005** |
| Current smoker n (%) | 0.772 | 0.434-1.374 | 0.379 |
| SBP (mmHg) | 1.022 | 1.002 – 1.043 | **0.029** |
| DBP (mmHg) | 1.033 | 1.007 – 1.060 | **0.012** |
| TG (mmol/L) | 0.710 | 0.570 –0.884 | **0.002** |
| TC (mmol/L) | 1.784 | 1.421 – 20240 | **＜0.001** |
| LDL (mmol/L) | 0.989 | 0.867-1.128 | 0.871 |
| HDL (mmol/L) | 0.084 | 0.033 – 0.214 | **＜0.001** |
| ALT (U/L) | 1.019 | 1.000-1.038 | 0.050 |
| AST (U/L) | 1.030 | 0.997-1.064 | 0.074 |
| GGT (U/L) | 1.023 | 1.008-1.039 | **0.003** |
| Uric acid (umol/L) | 1.007 | 1.004-1.011 | **＜0.001** |
| Creatinine (umol/L) | 1.030 | 1.007-1.053 | **0.011** |
| FBG (mmol/L) | 1.572 | 1.140-2.167 | **0.006** |
| ADAMTS13 (ug/mL) | 10.695 | 5.933-19.280 | **＜0.001** |

Dependent variable: OSA.

Abbreviations: OR, odds ratio; CI, confidence interval; BMI, body mass index; CAD, coronary artery disease; SBP, systolic blood pressure; DBP, diastolic blood pressure; TG, triglycerides; TC, total cholesterol; LDL, Low density lipoprotein; HDL, High density lipoprotein; ALT, alanine aminotransferase; AST, aspartate aminotransferase; GGT, g-glutamyl transferase; FBG, fasting blood glucose; OSA, Obstructive sleep apnea; ADAMTS13, a disintegrin and metalloprotease with a thrombospondin type 1 motif, member.

**Table S4** Multivariate linear regression analysis of circulating ADAMTS13 levels and AHI.

|  | Unadjusted |  |  | Model 1 |  |  | Model 2 |  |
| --- | --- | --- | --- | --- | --- | --- | --- | --- |
|  | B(95%CI) | *P* value |  | B(95%CI) | *P* value |  | B(95%CI) | *P* value |
| ADAMTS13(per ng/ml increase) | 9.618(7.087-12.149) | **＜0.001** |  | 8.569（5.989-11.149） | **＜0.001** |  | 7.335（4.285-10.385） | **＜0.001** |

Model 1: adjusted for age, sex, BMI, CAD and smoker.

Model 2: adjusted for Model 1 +SBP, DBP, TG, TC, LDL, HDL, ALT, AST, GGT, uric acid, creatinine and FBG.

CI, confidence interval; BMI, body mass index; CAD, coronary artery disease; DBP, diastolic blood pressure; SBP, systolic blood pressure; FBG, fasting blood glucose; GGT, g-glutamyl transferase; HDL, High density lipoprotein; LDL, Low density lipoprotein; LDL, low-density lipoprotein cholesterol; ALT, alanine aminotransferase; AST, aspartate aminotransferase; AHI, Apneahypopnea index; ADAMTS13, a disintegrin and metalloprotease with a thrombospondin type 1 motif, member.
